# Information technology systems in public sector health facilities in developing countries: the case of South Africa

**DOI:** 10.1186/1472-6947-13-13

**Published:** 2013-01-24

**Authors:** Gregory B Cline, John M Luiz

**Affiliations:** 1 Wits Business School, University of the Witwatersrand, PO Wits, Johannesburg, 2050, South Africa; 2 Graduate School of Business, University of Cape Town, Breakwater Campus, Portswood Road, Green Point, Cape Town, 8005, South Africa

**Keywords:** Hospital information systems, Information management, Electronic health records, Africa, South Africa

## Abstract

**Background:**

The public healthcare sector in developing countries faces many challenges including weak healthcare systems and under-resourced facilities that deliver poor outcomes relative to total healthcare expenditure. Global references demonstrate that information technology has the ability to assist in this regard through the automation of processes, thus reducing the inefficiencies of manually driven processes and lowering transaction costs. This study examines the impact of hospital information systems implementation on service delivery, user adoption and organisational culture within two hospital settings in South Africa.

**Methods:**

Ninety-four interviews with doctors, nurses and hospital administrators were conducted in two public sector tertiary healthcare facilities (in two provinces) to record end-user perceptions. Structured questionnaires were used to conduct the interviews with both qualitative and quantitative information.

**Results:**

Noteworthy differences were observed among the three sample groups of doctors, nurses and administrators as well as between our two hospital groups. The impact of automation in terms of cost and strategic value in public sector hospitals is shown to have yielded positive outcomes with regard to patient experience, hospital staff workflow enhancements, and overall morale in the workplace.

**Conclusion:**

The research provides insight into the reasons for investing in system automation, the associated outcomes, and organisational factors that impact the successful adoption of IT systems. In addition, it finds that sustainable success in these initiatives is as much a function of the technology as it is of the change management function that must accompany the system implementation.

## Background

Information Technology (IT) has the substantial potential to contribute to improving access to care, lowering overall costs, and streamlining operational efficiencies in the health system. Clinical automation and business process management are major global trends affecting both mature and developing healthcare markets. The motivation behind these trends lies in the potential to reduce the complexity of multiple legacy and paper-based systems, improve capacity of health systems to manage patients and their data, increase compliance with health regulations, ensure availability of information to support more efficient care, and enhance security around patient confidentiality [1,2]. In general, Hospital Information Systems (HIS) automate the patient administrative functions (such as patient profile information, scheduling of appointments, billing) and the clinical care functions (e.g. clinical notes, computerised prescriptions, online laboratory results, digital radiological imaging) and ultimately has the capability of eliminating paper processes within the clinical setting. This aims to create a more cost effective, resource efficient, informed health care service that can be accessed by all.

In emerging markets such as South Africa, primary and secondary clinics are often located in rural areas with poor road networks and interrupted services such as electricity and water. Manual paper-driven processes are relied upon for delivering patient care and fulfilling administrative tasks. Patient records are paper based, and health statistics are recorded in log books which are sent infrequently to a regional office for data capturing of metrics (e.g. infant mortality rates) into a centralized database [3]. In South Africa, the value of automation within the healthcare system is poorly understood as the investment in IT is often considered against the opportunity cost of improving basic infrastructure for the clinic, hiring additional health worker resources, or purchasing medicines or consumables required to improve access to care. However, the evidence is growing that in an economic environment of severe constraints the use of IT in health care has the ability to improve capacity and resource utilization precisely because it frees up other valuable inputs.

Notwithstanding concrete evidence proving that EHRs have the potential to improve workflow efficiencies and quality of medical care, the majority of health workers continue to follow manual processes within the clinical setting [4,5]. Simon et al. speculate that the success of new system integration into daily workflow is dependent on how effectively the workplace culture emphasizes quality and innovation, as well as the characteristics of the health workers involved, together with technology-related factors (in this regard, offices with EHRs were more likely to be using email, computerized scheduling systems and e-prescribing) [5].

Goldzweig et al. also studied the cultural barriers to system implementations in hospitals and confirmed that 77% of practices without an EHR are resistant to EHR systems, 72% of physicians believe that moving towards an electronic system will result in frequent downtime, 64% believe that the system will increase the physicians’ work time, and 60% fear that they do not have sufficient computer skills [6]. Despite all the cultural and organizational issues cited, the number one barrier noted by the authors was cost. The business case is a challenge, as it is not clear who benefits from the investment. One recommendation from the research is to pursue a model where the funders subsidize some of the costs as it is they who benefit substantially from the financial aspect, more so than the health providers or patients.

Littlejohns et al. found that introducing technology initially increased the workload for the clinicians, who were expected to adapt their workflow to the new systems without appreciating why they should commit additional effort to perform effectively the same job function [7]. This highlighted to the researchers the need to ensure that users understand the reasons for implementation from the beginning together with the complexity of the healthcare task that is being automated.

The South African public healthcare sector, like most developing countries, is burdened with many challenges, including the consequences of HIV/AIDS, tuberculosis and malaria; weak healthcare systems; under-resourced provider networks; and low staff morale. These challenges have translated into poor health outcomes relative to total health expenditure [8]. The key challenge facing the sector is inefficient distribution of resources, rather than lack of funding as South Africa’s total healthcare expenditure is higher than other countries of similar level of economic development. The aim of this research is to investigate how access to healthcare by large population bases can be improved through more efficient healthcare resource management through the automation of healthcare systems. To this end, the research examines the experience of HIS in two South African hospitals and the perceptions of stakeholders as to its effectiveness in introducing efficiencies into everyday processes. The paper identifies the need to invest in information systems as a required intervention in order to lower transactional costs, co-ordinate care, improve human resource management and measure improvements. Furthermore, it determines the systemic and workflow-related strategic and cost benefits that result from automating healthcare systems in South Africa.

## Methods

A mixed method approach was followed that consists of a quantitative approach to comparatively analyse user perceptions and experiences from various user groups, and a qualitative approach to unpack the reasoning behind user attitudes toward system automation. Structured interviews were conducted with all respondents, using a combination of closed-ended and open-ended questions, with three hospital population groups (nursing staff, doctors and hospital administrators). Results from the three user groups were collated and cross-checked to provide a richer, more complete understanding of system automation within public sector hospitals in South Africa.

The population consists of all doctors, nurses, and hospital administrators employed at Albert Luthuli Hospital (IALCH) in KwaZulu-Natal Province and Sebokeng Hospital in Gauteng Province. Both facilities are public sector hospitals that have implemented various components of automated systems. The research followed a non-probability sampling design and thus the sample is not representative of all doctors, nurses and hospital administrators working in the public healthcare sector. As there are fewer than five healthcare facilities that have effectively integrated automated workflow into daily business processes, the majority of the healthcare worker population in South Africa are familiar only with manual-based paper-driven processes. Respondents consisted of doctors, nurses and hospital administrators working in public sector hospitals where automated systems had already been implemented, as specific experience in the healthcare sector and with healthcare systems was required in order for these respondents to contribute meaningfully to the research. An initial pilot study was conducted to identify critical items that had been unintentionally omitted during the design of the research instrument. The results of these interviews were analysed for recurring issues and checked against the literature reviews to determine how best to strengthen the credibility of the questionnaire.

The research questions were designed to evaluate user perceptions relating to cost and efficiencies associated with Hospital Information Systems; perceptions pertaining to data confidentiality; the impact on patient treatment and service delivery; record management; the user experience; management’s support for system utilisation; provision of training; and willingness to adopt new systems. The aforementioned factors were identified as the determinants of sustainable system adoption between the three user groups. A total of 94 interviews were conducted (including three pilot study interviews) – 21 doctors, 41 nurses, 31 hospital administrators and an EMR implementation manager were interviewed. Ethical clearance for the research was obtained from Research Ethics Committee at the University of the Witwatersrand. The research instrument consists of a combination of a series of statements and open-ended questions. The first section of the questionnaire consists of questions relating to demographic data of the respondent. The second section of the questionnaire consists of 41 statements using a Likert scale relating to various system technologies that are part of daily workflow; the impact of automation in terms of cost and strategic value in healthcare facilities; and the degree to which organizational culture influences the implementation of new workflows and processes within the hospital (see Additional file 1). Ordinal data obtained from the survey questionnaire was classified and subjected to analysis according to a Likert scale consisting of seven response categories plus a category for responses where the answer was not known. A distribution-fitting approach was applied in order to analyse the collected data and categorize the various criteria [9]. The third section of the questionnaire consists of seven open-ended questions used to assess the qualitative factors associated with automated workflow and perceived benefits or pitfalls. The questionnaire covered five broad areas: questions relating to respondents’ experiences and perceptions before system installation; respondents’ experiences and perceptions after system installation; organisational influences relating to end-user adoption; barriers to effective functioning of the system; and the overall satisfaction with the computerised information system.

The interviews were conducted face-to-face with respondents in February 2011 and captured using a mobile phone-based research software application for data collection and recordings. Health Systems Trust (HST), an independent non-government organisation with extensive expertise in South African public sector health systems research, assisted with data collection at the two sites. HST performs research on behalf of the South African Department of Health and World Health Organisation amongst other local and global health organisations. Three HST researchers were trained on the research instrument before travelling to the two sites. All the researchers conducted interviews together for the first day at Sebokeng Hospital (2 February, 2011) and at IALCH (8 February) to ensure that individually they would all subsequently follow a consistent protocol to the interviews. This was part of the process through which we ensured the reliability of the results throughout the process which is further discussed below.

### Validity and reliability

To ensure that different perspectives were obtained for the research, the sample base was drawn from healthcare provider/administrator populations in public facilities, across two provinces and was indiscriminate of race or gender. Drawn samples consisted of doctors, nurses, and healthcare administrators who had varied experience in interacting with healthcare systems at the designated facility. Owing to the limited number of automated systems in public sector hospitals in South Africa, samples were drawn from the two available facilities that had already implemented systems to some degree.

Steps taken to improve external validity during the study consisted of drawing a large pool of respondents who had adapted to an information system-based workflow for at least one year. The healthcare professionals employed at the chosen hospital sites were expected to be familiar with the challenges and limitations of healthcare delivery in the public sector. XRespondents were drawn from at least two provinces, Gauteng and KwaZulu-Natal, but were limited to healthcare facilities where components of information systems had been implemented (see section below for a full discussion of the HIS components that were implemented). To further ensure consistency, a mixed method approach was followed to consolidate qualitative feedback with the quantitative survey data.

To ensure that internal validity was preserved, respondents were considered according to three population groupings: doctors, nurses and administrators. A pilot study was conducted to ensure that the research instrument questionnaire was easy to understand and elicited the intended information from respondents. The research instrument was standardised and consistent for each group of respondents.

To ensure reliability of the study, a standardised research tool was developed (see Additional file 1) and administered face-to-face in a consistent manner with all respondents such that the steps followed in the research could be repeated in a duplicate study. The questions included in the survey focused on the personal experience and perceptions of each respondent. Interviews were conducted in privacy, with care taken to ensure that respondents did not influence answers amongst themselves.

Methodologically, given the nature of this research, it presents certain limitations. As information systems have been implemented in a small number of health facilities in South Africa, a narrow number of respondents qualified for the study. Furthermore, the research may have been exposed to biased selection techniques as public sector employees may have various reasons for participating or not participating in the study. The relationship between public sector employees and the state can be described as tenuous, as health officials have in the past been disciplined for speaking negatively regarding the Department of Health in the public domain. While the sample is non-random, the standardisation of interpretation of results assumes that the frequency of responses reflects the import of what is being measured. To minimise this risk, a wide spectrum of respondents within the two sites was selected.

## Results and discussion

IALCH is an 840-bed tertiary facility in the city of Durban and is one of the few digital hospitals in the country. Sebokeng Hospital is a 704-bed regional hospital with three associated clinics. At both hospitals AME International is responsible for implementing the complete HIS which consists of:

Electronic Medical Records;

Picture Archiving and Communication System;

Radiological Information System;

Laboratory Information System;

Pharmacy Information System;

Critical Care System;

Administration and Financial Systems; and

Human Resource Management System.

Patient Management and Scheduling;

Clinical Documentation;

Visit Summaries and Discharge Summaries; and

Statistics and Reporting.

The discussion below indicates that respondents from both hospitals agreed that the HIS has the potential to impact positively on information security, workflow optimization, cost reduction and patient care.

### Perceptions of manual processes

The research data confirms that for hospital staff conditioned to working in non-automated environments, the perceptions of inefficiency relating to paper-driven processes are not a major factor. The preponderance of doctors disagreed with the statement that paper processes resulted in more of their time being dedicated to administrative activities. This is likely attributable to the fact that doctors did not perceive the urgency of making detailed clinical notes as they are less under threat of being sued for medical liability than their global colleagues. This is because, while the notion of punitive damages for medical liability in South Africa exists, in general these damages are not recoverable. Thus, it could be expected that it would be nurses and staff who are delegated a high administrative workload that would hold this perception. In addition, 71% of the nursing sample group recognized the need for duplicate data entries, which is to be expected, given that nurses are tasked with recording multiple aspects of patient data on a frequent basis, e.g. patient history, medication, observations, and laboratory results.

The observations suggest that paper processes are not as inefficient as is suggested by the literature [6]. Rather, the physician resistance to adoption was demonstrated (with 55% of doctors believing that paper processes were efficient) through the unwillingness to adopt new workflow processes that required data to be accessed and entered onto a computer system. This is in keeping with the findings of Poon et al. which suggested that the number one barrier to adoption resides in the change management function, which needs to be addressed in order to shift perceptions towards acceptance of HIS [10].

### Perceptions of hospital information systems

While respondents recognize that there are benefits to be gained from automation, the direct impact on their daily business processes is not well understood. Some staff perceived the introduction of hospital systems as disruptive to patient care, with one nurse from Sebokeng Hospital commenting that “concerning patient care on the nursing side, more time is now taken to sit in front of a computer instead of taking care of the patient”. Thus, obtaining buy-in and acceptance for the implementation of a computerized system from hospital staff should not rely on broad promises of potential optimization benefits, but should rather target processes specific to the various user groups within their daily workflow that are genuinely perceived as inefficient.

Table 1 describes the data relating to the impact of automation and the perceived barriers to adoption for doctors, nurses and hospital administrators, respectively. Using the distribution fitting algorithm a positive and statistically significant t-value indicates that the factor is statistically more important relative to the other factors and that there is more agreement with the factor or statement, a negative and significant t-value indicates the factor is less important and that there is less agreement with the factor [9].

**Table 1:** Perceptions of hospital information systems – doctors, nurses and administrators

**Doctors**	**Absolutely disagree**	**Strongly disagree**	**Somewhat disagree**	**Neither agree nor disagree**	**Somewhat agree**	**Strongly agree**	**Absolutely Agree**	**Total**	**t-value**	**p-value**	**Significance**
I find it easier to work with electronic system than with paper records	0	0	2	4	4	6	4	20	2.881	0.0098	+
I have confidence that information is more secure and confidential in electronic compared to paper	1	4	6	1	0	6	1	19	-0.4984	0.6246	
I believe the computer systems will save the hospital money	1	2	2	3	3	5	1	17	0.4029	0.6927	
I believe the cost of learning new computer systems is not wort the benefits	4	7	4	0	3	1	0	19	-4.2001	0.0006	-
I prefer using a paper based system	4	7	4	2	2	1	0	20	-4.6680	0.0002	-
I would like to move to a paperless system as soon as possible	1	1	1	3	2	3	9	20	2.9370	0.0088	+
**Nurses**											
I find it easier to work with electronic system than with paper recors	1	2	2	1	9	15	10	40	3.6251	0.0008	+
I have confidence that information is more secure and confidential in electronic compared to paper	3	1	3	3	4	18	8	40	2.0762	0.0447	+
I believe the computer system will save the hospital money	1	1	1	3	10	11	4	31	1.5837	0.1241	
I believe the cost of learning new computer systems is not worth the benefits	9	14	4	2	3	5	0	37	-5.9191	0.0000	-
I prefer using a paper based system	13	14	4	3	2	1	3	40	-4.9693	0.0000	-
I would like to move to a paperless system as soon as possible	1	2	0	2	4	18	13	40	4.7026	0.0000	+
**Administrators**											
I find it easier to work with electronic system than with paper recors	1	1	0	1	0	17	10	30	3.6777	0.0010	+
I have confidence that information is more secure and confidential in electronic compared to paper	1	3	0	2	2	12	10	30	3.1072	0.0043	+
I believe the computer systems will save the hospital money	0	2	1	5	3	9	7	27	2.5319	0.0180	+
I believe the cost of learning new computer systems is not worth the benefits	7	14	1	3	1	2	2	30	-4.5328	0.0001	-
I prefer using a paper based system	13	16	0	1	0	0	0	30	-16.0300	0.0000	-
I would like to move to a paperless system as soon as possible	4	0	0	0	2	12	12	30	2.0536	0.0495	+

For all three sample groups (doctors, nurses and administrators) that had experience using the HIS, there was consensus that it was easier to work with electronic systems compared to paper records. The data also revealed a belief across all three sample groups that the cost of learning the new system was worth the benefits, that the HIS was preferred to the previous paper-based system, and that hospital staff would like to move to the HIS for all functions as soon as possible.

The benefits to doctors include superior access to patient record information and the ability to provide superior care based on better informed decision making. The literature review component of the research identified the benefits to doctors as improved patient assessments, treatment plans and clinical trials. While improved service quality and better care outcomes are anticipated, the research did not seek to understand the direct benefit to doctors as they were implicitly understood as a desirable outcome of automated systems.

### Information security and patient confidentiality

Over 75% of nursing staff and hospital administrators were confident that information is more secure and confidential in electronic format compared to a paper-based system. While this was a statistically significant factor for these sample groups, 58% of doctors surveyed disagreed. Qualitative data provided some insight, with one doctor at IALCH confirming that staff can access data of patients that are not directly in their care and that “people who don’t work in that department should not be able to access records of patients in other departments”. As described in the literature review, privacy and confidentiality of data are fundamental concerns with international HIS implementations [5]. There is currently no protocol or guidance in South Africa as to the exact measures that should be implemented to protect patient confidentiality effectively. It can be inferred that electronic records have the ability to offer password-restricted access to electronic patient information; however, hospital staff was discouraged by the fact that once a user logs onto the system, that person has complete access to all patient records regardless of who the treating doctor is, or which ward the patient is allocated to. Going forward, measures should be taken to ensure that security and privacy of information are included in the system security and design architecture in accordance with the hospital risk management function.

### Lowering costs

As to the perceived costs and benefits of implementing the new system, the statement “I believe the hospital will save costs as a result of moving to the new system” was identified as statistically significant by nurses and administrators but not by doctors. From the qualitative interviews, it was clear that while nurses and administrators believed that IT would reduce costs, both sample groups did not understand how this would be achieved. When asked “Do you see cost savings as a result of the system?”, one nurse at IALCH replied “Yes, it saves time; more patients are admitted because it is faster than writing.” One possible explanation is that the positive attitude towards the IT system within these two sample groups translated into a logical association of workflow efficiencies with cost reduction. One doctor felt: “It’s costing the hospital as well as provincial government a lot of money. The cost of running a computer system is expensive. You need to have someone to maintain the system on call 24 hours a day.” Another clinician was of the opinion that “The hospital will save money as they don’t have to buy as much paper. Information will be accessed more efficiently and patients will be treated more efficiently, so there’s a faster turnaround time. For example, to discharge a patient, it’s all done on the system and the patient just goes to one place and all paperwork is done there.”

Overall the perceived reasons that influence the investment of automated systems within a hospital are the ability to reduce administrative workload associated with data capture; the optimization of workflow; the potential to enhance patient data security; and the potential for lower overall costs. The research suggests that there is an opportunity to include the IT value proposition specific to cost reduction in the change management process during system implementation (and education of doctors, nurses and administrators) in order to gain further support for system adoption and usage.

### Outcomes experienced from automating systems in hospital departments

Following the implementation of the hospital system, respondents were asked about their perceptions relating to the impact of automation in terms of cost and strategic value of the system. The results provided insight into the impact of automation on service delivery and hospital reputation perceptions; patient record management; and HIS user experience (see Table 2).

**Table 2:** Service delivery and hospital reputation perceptions – doctors, nurses and administrators

**Doctors**	**Absolutely disagree**	**Strongly disagree**	**Somewhat disagree**	**Neither agree nor disagree**	**Somewhat agree**	**Strongly agree**	**Absolutely Agree**	**Total**	**t-value**	**p-value**	**Significance**
Patient waiting times for admissions have decrease	2	3	1	3	3	6	0	18	-0.0914	0.9283	
Patient waiting times to be seen by a doctor or nurse are decreased	1	4	2	0	6	4	0	17	-0.4756	0.6412	
Patient overall satisfaction with care received is higher	1	2	1	3	4	5	2	18	0.9382	0.3621	
I am treating more patients per shift in the outpatient/ward/where I work	2	4	2	3	4	4	1	20	-0.7749	0.4484	
There is increased satisfaction with the overall working conditions in the hospital	1	1	1	3	5	6	3	20	1.7130	0.1039	
The hospital has enjoyed improved service delivery	1	2	1	3	1	9	2	19	1.6998	0.1074	
**Nurses**											
Patient waiting times for admissions have decrease	0	4	3	3	4	15	8	37	2.7761	0.0088	+
Patient waiting times to be seen by a doctor or nurse are decreased	1	0	1	5	10	17	4	38	1.9725	0.0563	
Patient overall satisfaction with care received is higher	1	0	1	1	12	18	6	39	2.9657	0.0053	+
I am treating more patients per shift in the outpatient/ward/where I work	0	3	2	6	8	10	8	37	2.1513	0.0384	+
There is increased satisfaction with the overall working conditions in the hospital	0	1	7	2	9	14	7	40	2.2247	0.0321	+
The hospital has enjoyed improved service delivery	1	1	2	1	12	15	6	38	2.6109	0.0131	+
**Administrators**											
Patient waiting times for admissions have decrease	1	1	1	2	2	10	8	25	2.6938	0.0130	+
Patient waiting times to be seen by a doctor or nurse are decreased	0	1	1	2	3	11	5	23	3.3013	0.0034	+
Patient overall satisfaction with care received is higher	0	0	0	5	5	10	2	22	2.1914	0.0404	+
I believe more patients are treated per shift as a result of the system	0	1	0	2	2	9	10	24	4.8093	0.0001	+
There is increased satisfaction with the overall working conditions in the hospital	0	3	0	1	4	10	10	28	3.5554	0.0015	+
The hospital has enjoyed improved service delivery	0	1	0	2	2	18	6	29	5.3258	0.0000	+

### Impact on service delivery and hospital reputation perceptions

As was to be expected, the overall perceptions relating to the impact on patient experience were overtly positive. For example, at IALCH 86% of respondents stated that patient waiting times to be seen by a doctor or nurse had decreased, whilst 66% maintained that at Sebokeng. Significant statistical factors were observed with nursing and administrator respondents regarding patient waiting times for admission and discharge, and overall patient satisfaction with care received. It stands to reason that as nursing staff and administrators are involved in the administrative tasks relating to patient admission and discharge, the perception of these two sample groups would hold a higher level of implication concerning where automation can replace manual processes. Doctors on the other hand did not identify these factors relating to patient workflow as noteworthy, as they are not involved in patient administrative tasks and the system was not perceived to enhance the doctors’ ability to treat patients more effectively.

Given that patients spend more time with nurses and administrators during the admission, waiting and discharge processes compared to the time spent with the doctor for consultation and treatment, it is likely that the nurses and administrators perceive the hospital to have experienced a higher level of service delivery to patients as a result of the automated system. One nurse at Sebokeng Hospital commented: “waiting time decreased … You don’t have to look for papers to file, staple or punch.” Research findings were consistent with those of Poon et al.’s confirming that positive patient experience and hospital reputation perceptions are supported through IT investment [10].

The image of public sector tertiary hospitals in South Africa has been an emotive issue. Public opinion considers these facilities to be antiquated, under resourced and associated with poor service delivery. The results of this study suggest not only that IT has a role to play in modernizing state hospitals in terms of automation but that there is a real belief that automated systems are an important component to improving health service delivery, patient experience and the overall public perception of state healthcare services. IT systems also have a role to play in the reputational management of internal stakeholders, as evidenced by the perceived increased satisfaction in overall working conditions within the hospital, particularly for 87% of nurses and 86% of administrators surveyed.

### Patient record management

The majority of hospital staff was in agreement as to the benefits of EMRs (see Table 3).

**Table 3:** Electronic health record perceptions – doctors, nurses and administrators

**Doctors**	**Absolutely disagree**	**Strongly disagree**	**Somewhat disagree**	**Neither agree nor disagree**	**Somewhat agree**	**Strongly agree**	**Absolutely Agree**	**Total**	**t-value**	**p-value**	**Significance**
I have superior access to patient record information when compared to paper based systems	0	2	1	1	2	3	11	20	3.6665	0.0018	+
There is a reduction of duplication of information which means cleaner patient records and less time spent entering information	2	1	4	0	3	8	2	20	0.7483	0.4640	
Patient information is more disorganised with the computer system compared to the paper system	4	9	2	1	1	2	1	20	-3.1743	0.0053	-
Fewer records are lost and record management has improved	0	0	0	0	6	6	8	20	5.9135	0.0000	+
**Nurses**											
I have superior access to patient record information when compared to paper based systems	1	0	0	0	8	16	13	38	4.6403	0.0000	+
There is a reduction of duplication of information which means cleaner patient records and less time spent entering information	2	0	1	2	12	15	7	39	2.0770	0.0448	+
Patient information is more disorganised with the computer system compared to the paper system	10	19	6	2	1	0	2	40	-5.8010	0.0000	-
Fewer records are lost and record management has improved	2	4	1	0	4	18	10	39	2.9957	0.0049	+
**Administrators**											
I have superior access to patient record information when compared to paper based systems	1	0	0	1	3	16	8	29	3.1605	0.0039	+
There is a reduction of duplication of information which means cleaner patient records and less time spent entering information	1	3	2	2	3	14	5	30	1.6292	0.1145	
Patient information is more disorganised with the computer system compared to the paper system	10	11	3	2	1	2	1	30	-5.8357	0.0000	-
Fewer records are lost and record management has improved	0	2	1	4	3	10	9	29	3.3449	0.0024	+

Doctors, nurses and administrators all identified the statement “I have superior access to patient record information when compared to paper based systems” as being very important relative to other factors with t-values of 3.66, 4.64 and 3.16 respectively. A doctor from IALCH confirmed “Patient care is improved because access to results are easier and don’t get lost. Results are logged and you can prove that you ordered them.” The perceived advantages of EMRs were shared by the nursing respondents. As a nurse from IALCH commented: “The system is user friendly, very fast, and saves money because the doctors are able to read the scans from the rural hospitals without the patient, the decision is made on the phone, results are posted through the computer, multidisciplinary teams are able to work fast with the system and patient care is improved.” The research suggests a unanimous recognition of the value of HIS and raises questions as to how this capability will enhance the clinician’s ability to improve healthcare delivery. Nurses and administrators believe that the HIS results in fewer records lost, improved record management, and the reduction of duplication of information. A nurse at IALCH confirmed: “Record keeping is good as the history of the patient is written on the computer and is well kept and can be retrieved easily.”

### Hospital information system user experience

The HIS user experience varied between the three sample groups. Doctors, nurses and administrators argued that the system was easy to use with 87% disagreeing with the statement that they found the system difficult to use. But only nurses and administrators believed the statement “I find the computerized system is faster and easier to use compared to handwritten notes” to be very important relative to other factors (78% of nurses and 80% of administrators believed that the fact that computerized systems were faster than handwritten notes was an important factor while only 55% of doctors agreed with this statement to varying degrees). Opinion as to whether the users perceived the system as slow varied, with doctors emerging as the most sensitive to system performance. The reasons relating to system usability and performance were not well understood, and while infrastructure and system design considerations are not evaluated in this study, it can be inferred that these factors have the potential to define the user experience and hence the user attitude towards system adoption and usage.

Respondents indicated that they were eager to use the new system, were comfortable using a computer, and believed that they could not get by without having to learn a computer system. However, their responses indicating their willingness to use the computer; the impact of the system on morale; and their perceptions of how computers convey professionalism in the hospital environment varied (see Table 4).

**Table 4:** Willingness to adopt new systems – doctors, nurses and administrators

**Doctors**	**Absolutely disagree**	**Strongly disagree**	**Somewhat disagree**	**Neither agree nor disagree**	**Somewhat agree**	**Strongly agree**	**Absolutely Agree**	**Total**	**t-value**	**p-value**	**Significance**
Staff are eager to learn the new system	1	2	3	2	1	7	3	19	1.3622	0.1909	
Initially I did not want to move to the computer system	6	6	2	4	2	0	0	20	-4.8408	0.0001	-
Initially the hospital staff did not want to move to the computer system	0	2	1	3	5	2	0	13	-0.1996	0.8454	
As a result of the computer systems, I see improved moral in the workplace	2	2	0	6	4	1	2	17	-0.3466	0.7337	
As a result of the computer systems, my overall level of professionalism is increased	2	2	1	5	5	3	2	20	-0.0862	0.9322	
I have a fear of having to use a computer instead of a paper	9	8	0	3	0	0	0	20	-7.0867	0.0000	-
I can get by without having to learn the computer system	5	7	1	0	2	1	0	16	-4.3597	0.0007	-
**Nurses**											
Staff are eager to learn the new system	3	0	3	1	10	14	9	40	1.9387	0.0600	
Initially I did not want to move to the computer system	7	15	2	0	4	6	6	40	-2.3553	0.0238	-
Initially the hospital staff did not want to move to the computer system	0	6	4	3	4	9	4	30	0.2093	0.8357	
As a result of the computer systems, I see improved moral in the workplace	2	1	6	5	7	14	4	39	0.5032	0.6178	
As a result of the computer systems, my overall level of professionalism is increased	0	2	5	1	8	13	11	40	3.4205	0.0015	+
I have a fear of having to use a computer instead of a paper	14	20	1	1	3	1	0	40	-11.2977	0.0000	-
I can get by without having to learn the computer system	15	16	4	0	1	3	1	40	-7.2398	0.0000	-
**Administrators**											
Staff are eager to learn the new system	0	3	0	2	2	16	7	30	3.6346	0.0011	+
Initially I did not want to move to the computer system	5	17	0	1	0	3	2	28	-4.2944	0.0002	-
Initially the hospital staff did not want to move to the computer system	0	10	1	2	1	3	3	20	-1.6708	0.1121	
As a result of the computer systems, I see improved moral in the workplace	1	2	1	1	4	11	10	30	3.0791	0.0046	+
As a result of the computer systems, my overall level of professionalism is increased	2	0	0	2	4	10	12	30	2.8525	0.0081	+
I have a fear of having to use a computer instead of a paper	10	19	0	1	0	0	0	30	-16.8528	0.0000	-
I can get by without having to learn the computer system	13	12	1	0	1	1	0	28	-9.2708	0.0000	-

Respondents were asked about their perceptions regarding the impact of the new computer system on morale in the workplace. While doctors did not see a link between hospital morale and the new computer system, 64% of nurses agreed that there was a positive relationship, and administrators perceived the relationship as statistically significant with a t-value of 3.63. The results are likely a reflection of the degree to which the three sample groups depend on computer systems for their job functions. There were, however, varying opinions, with one of the doctors at IALCH expressing the opinion that the system “lifts morale because you are living in a very modern environment; access to patient information is quick, less work and saves time; there is improved communication between departments”.

Nurses and administrators identified ‘the ability of systems to increase individual levels of professionalism’ as being very important relative to other factors, while doctors did not find this statement of statistical importance. A nurse at Sebokeng Hospital commented: “I think the presence of the electronics in our work has improved confidence and morale, and it puts the hospital at a certain level; once you come in and see your information has been computerized you will quickly develop that confidence in the future.”

## Conclusion

This study examined the impact of hospital information systems implementation on service delivery, user adoption and organisational culture within two hospital settings in South Africa. Important differences in terms of perceptions were observed among the three sample groups of doctors, nurses and administrators as well as between our two hospital groups. Respondents indicated a positive influence of automation in terms of cost and strategic value in public sector hospitals with regard to patient experience, hospital staff workflow enhancements, and overall morale in the workplace. The research provides insight into the reasons for investing in system automation, the associated outcomes, and organisational factors that impact the successful adoption of IT systems. In addition, based upon the experience of our respondents, we find that the benefits of the system are only fully understood and appreciated once all components are implemented and holistic change management support has been applied. The research highlights a number of areas where stakeholders’ perceptions of the HIS indicated room for improvement through a change management process. Suggested actions would include: a) identifying and targeting usage models for workflow optimization specific to each user group as user experience differ between doctors, nurses and administrators; b) providing potential users with exposure to the HIS or demonstrations to assist with internalizing automation benefits and thereby driving system adoption; c) enhancing patient confidentially within the system as this was an area of concern expressed by respondents; and d) developing a value proposition specific to each user group to encourage support, adoption and usage of the new system.

Given the demands facing the public healthcare sector in developing countries relating to resource allocation and utilization, access to healthcare services, and efficient management of patient encounters at tertiary public health facilities, the research suggests the important impact of IT in ameliorating these challenges. Automation of systems and processes complements the evolution of patient treatment protocols, medical record management, and back office administrative functions while enabling time and cost savings within the healthcare system. Health systems have the opportunity to alleviate some of their resource constraints and reduce transactional costs by investing in technology to help better co-ordinate care and move all functions of public health management into the service economy.

While the study was considered against the backdrop of South Africa, the research was contextualised within countries that shared common attributes such as similar social, economic and physical infrastructure. The reasons for investing, the impact of automation and the organisational influences affecting successful implementations were considered in the literature review against similarities found in other emerging countries including Nigeria, Kenya, China, Brazil and India. The common factors influencing these countries lie in the lack of basic infrastructure that impedes workflow and the investment required to bring health services to a level of parity with developed nations. Thus the findings of the study should be equally applicable to other emerging countries sharing similar challenges.

### Consent

Ethical clearance for the research was obtained from Research Ethics Committee at the University of the Witwatersrand. Written informed consent was obtained from the two hospitals for publication of this case report. A copy of the written consent is available for review by the Editor-in-Chief of this journal.

## Competing interest

The authors have no conflict of interest.

## Authors’ contribution

Both authors were involved in the study conceptualization, design and data interpretation as well as the manuscript preparation. GC was responsible for the data acquisition. JL was responsible for the approval of the final version of the paper to be submitted.

## Pre-publication history

The pre-publication history for this paper can be accessed here:

http://www.biomedcentral.com/1472-6947/13/13/prepub

## Supplementary Material

Additional file 1 **Appendix A.** Extract from Questionnaire Instrument (Likert Scale Structured Questions). Please answer the following questions by indicating which answer most accurately represents the extent to which you agree or disagree with the statement on the left. There can only be one answer per statement. Click here for file
