# Supplementary material for: Information technology systems in public sector health facilities in developing countries: the case of South Africa
Source: BMC Med Inform Decis Mak. 2013 Jan 24;13:13. doi: 10.1186/1472-6947-13-13 (PMC3570341; doi:10.1186/1472-6947-13-13)

**Appendix A: Extract from Questionnaire Instrument (Likert Scale Structured Questions)**

Please answer the following questions by indicating which answer most accurately represents the extent to which you agree or disagree with the statement on the left. There can only be one answer per statement.


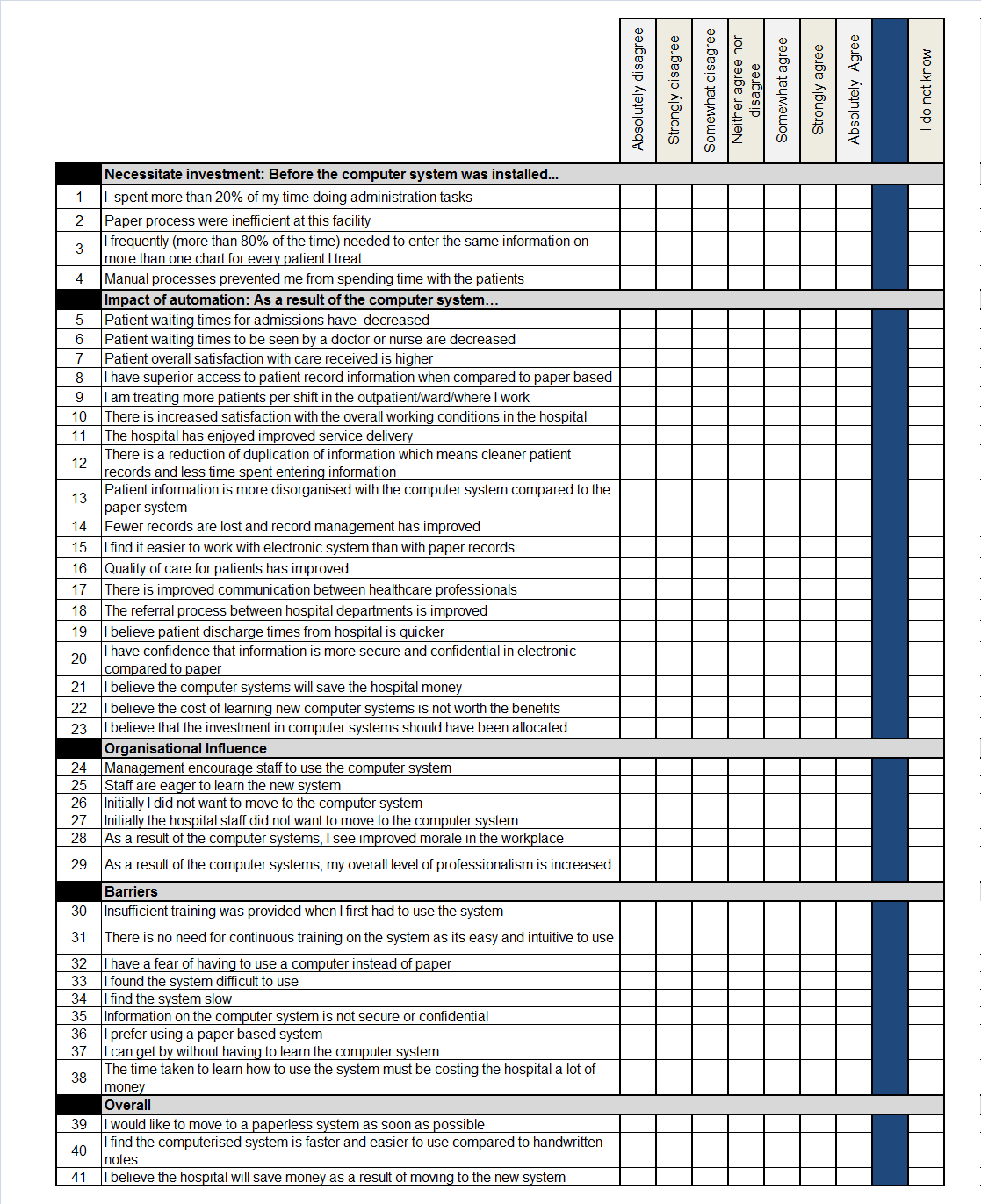

Supplement: Additional file 1 — Appendix A. Extract from Questionnaire Instrument (Likert Scale Structured Questions). Please answer the following questions by indicating which answer most accurately represents the extent to which you agree or disagree with the statement on the left. There can only be one answer per statement. [file 1472-6947-13-13-S1.doc]
